# Supplementary material for: Dynamic co-evolution of transposable elements and the piRNA pathway in African cichlid fishes
Source: Genome Biol. 2025 Jan 22;26:14. doi: 10.1186/s13059-025-03475-z (PMC11753138; doi:10.1186/s13059-025-03475-z)
Supplement: Supplementary file 1 — Additional file 1. Supplementary figures: Figs S1-S11. [file 13059_2025_3475_MOESM1_ESM.pdf]

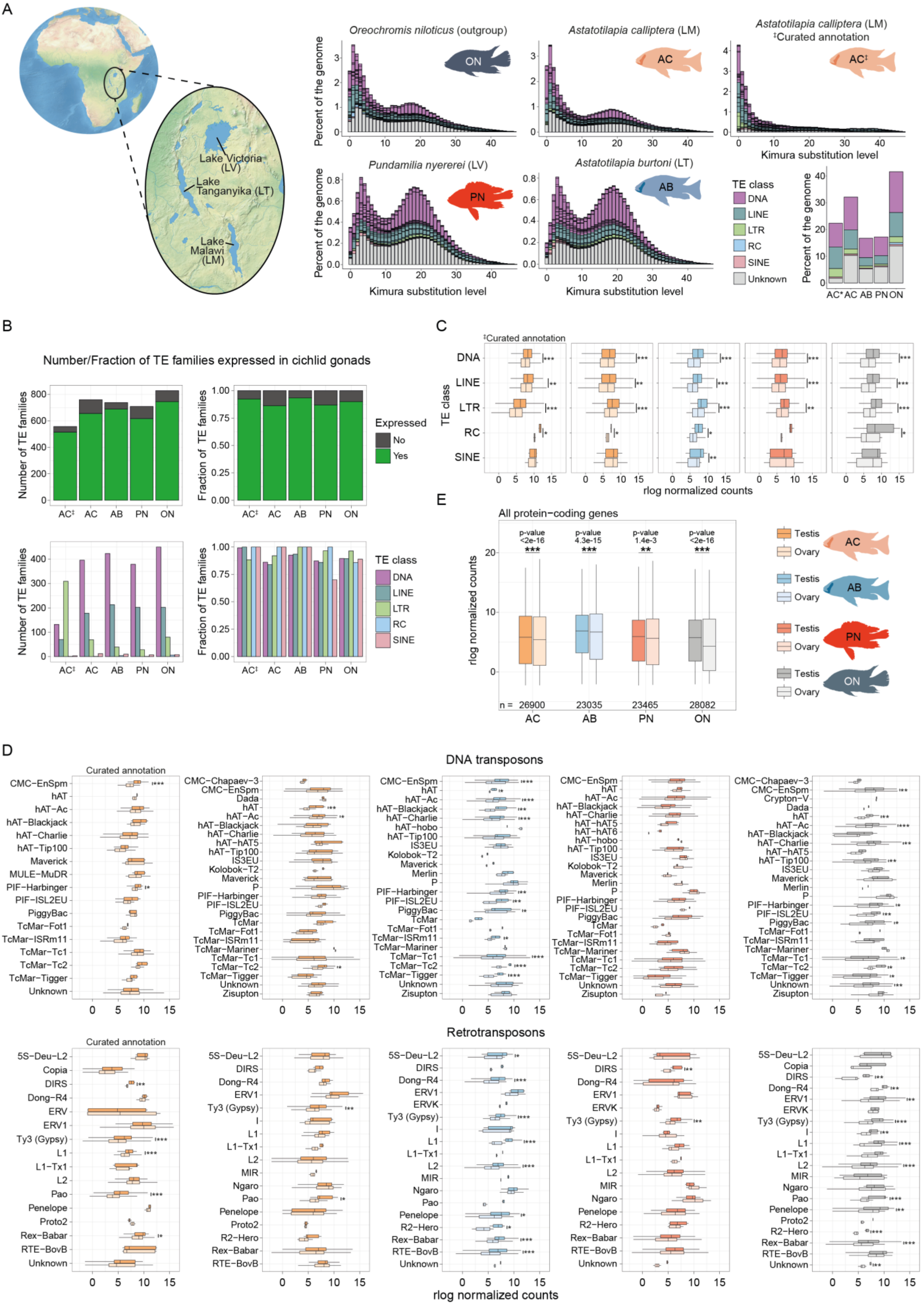
A
Lake Victoria (LV)
Lake Tanganyika (LT)
Lake Malawi (LM)
Oreochromis niloticus (outgroup)
Astatotilapia calliptera (LM)
Astatotilapia calliptera (LM) ‡Curated annotation
Pundamilia nyererei (LV)
Astatotilapia burtoni (LT)
ON
AC
AC‡
PN
AB
Percent of the genome
Kimura substitution level
TE class
DNA
LINE
LTR
RC
SINE
Unknown
AC* AC AB PN ON
B
Number/Fraction of TE families expressed in cichlid gonads
Number of TE families
Fraction of TE families
Expressed
No
Yes
AC‡ AC AB PN ON
C
‡Curated annotation
TE class
rlog normalized counts
E
All protein−coding genes
p-value <2e-16
p-value 4.3e-15
p-value 1.4e-3
p-value <2e-16
rlog normalized counts
n = 26900
23035
23465
28082
Testis
Ovary
D
DNA transposons
Curated annotation
Retrotransposons
rlog normalized counts

Fig. S1

Fig. S1. The TE landscape and expression dynamics in East African cichlid fishes. (**A**) Illustration of the East African Great Lakes as shown in **Figure 1A** and plots depicting the TE landscape of the representative species used in this study. The CpG-adjusted Kimura substitution level is the divergence from a consensus TE modelled for each family. All major TE classes are shown, as well as repeats classified as unknown. For *Astatotilapia calliptera*, the Lake Malawi representative, we use two TE annotations, one of which was curated. The second burst of TE expansion is more prominent in the *P. nyererei* and *A. burtoni* genomes, which have the most fragmented genomes. *O. niloticus* and *A. calliptera* have chromosome-level genome assemblies that display a relatively reduced second burst of TE expansion. Thus, it is likely that genome fragmentation affected TE modelling and TE annotation. A stringently curated TE library (see **Methods**) improved the annotation of younger TE families. Bar plot on the bottom right compiles the percent of the genome occupied by each TE class in each of the representative cichlid species. Maps obtained from Natural Earth, naturalearthdata.com. (**B**) Number of TE families expressed in cichlid gonads. First two panels depict overall numbers or proportion of expressed families, while the panels below correspond to the number and proportion of expressed TE families by TE class. (**C**) Expression of TE families in cichlid gonads grouped by TE class, shown as regularised log (rlog) normalised counts. P-values were calculated with Wilcoxon rank-sum tests (using Benjamini & Hochberg correction) comparing expression in ovaries and testes for each TE class. (**D**) Expression of TE families in cichlid gonads grouped by TE superfamily. Panels above depict DNA transposon superfamilies and panels below depict retrotransposon superfamilies. Expression shown as rlog normalised counts. P-values were calculated with Wilcoxon rank-sum tests (using Benjamini & Hochberg correction) comparing expression in ovaries and testes for each TE superfamily. Same colour code by species and organ as in (C). (**E**) Overall expression levels, in rlog normalised counts, of all protein-coding genes in each species in testes versus ovaries. The number of annotated protein-coding genes used for this analysis is indicated above the x axis. P-values were calculated with Wilcoxon rank-sum tests (using Benjamini & Hochberg correction) comparing overall expression in ovaries and testes. (C-E) Significance notation as follows: *$0.01 \leq p$ value < 0.05; **$0.001 \leq p$ value < 0.01; *** p-value < 0.001. AB, *Astatotilapia burtoni*; AC, *Astatotilapia calliptera*; LM, Lake Malawi; LT, Lake Tanganyika; LV, Lake Victoria; ON, *Oreochromis niloticus*; PN, *Pundamilia nyererei*; rlog, regularised log.

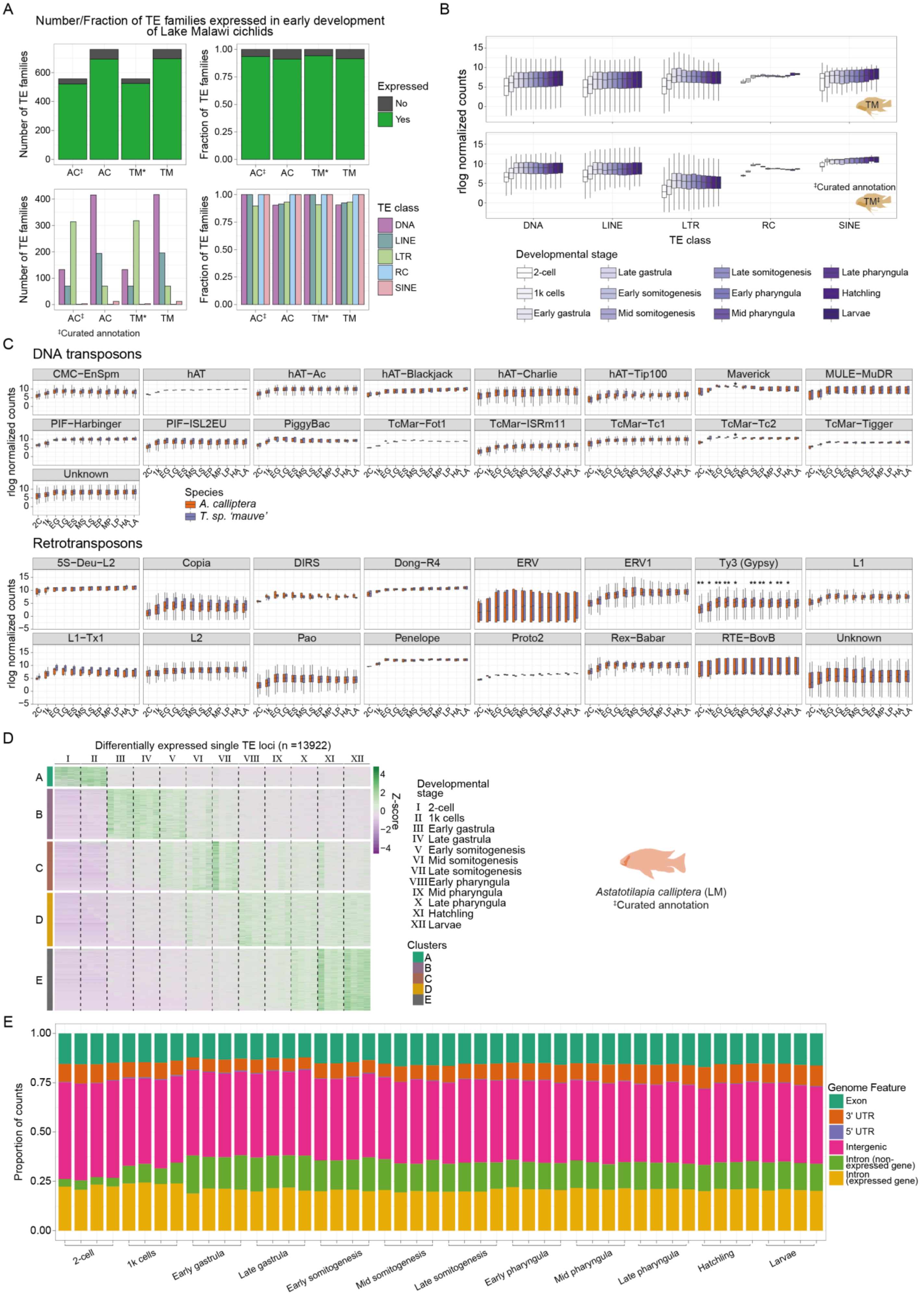
A
Number/Fraction of TE families expressed in early development of Lake Malawi cichlids
Number of TE families
Fraction of TE families
AC‡
AC
TM*
TM
Expressed
No
Yes
TE class
DNA
LINE
LTR
RC
SINE
‡Curated annotation
B
rlog normalized counts
TE class
TM
‡Curated annotation
TM‡
Developmental stage
2-cell
1k cells
Early gastrula
Late gastrula
Early somitogenesis
Mid somitogenesis
Late somitogenesis
Early pharyngula
Mid pharyngula
Late pharyngula
Hatchling
Larvae
C
DNA transposons
CMC−EnSpm
hAT
hAT−Ac
hAT−Blackjack
hAT−Charlie
hAT−Tip100
Maverick
MULE−MuDR
PIF−Harbinger
PIF−ISL2EU
PiggyBac
TcMar−Fot1
TcMar−ISRm11
TcMar−Tc1
TcMar−Tc2
TcMar−Tigger
Unknown
Species
A. calliptera
T. sp. 'mauve'
Retrotransposons
5S−Deu−L2
Copia
DIRS
Dong−R4
ERV
ERV1
Ty3 (Gypsy)
L1
L1−Tx1
L2
Pao
Penelope
Proto2
Rex−Babar
RTE−BovB
Unknown
D
Differentially expressed single TE loci (n =13922)
Z-score
Developmental stage
I 2-cell
II 1k cells
III Early gastrula
IV Late gastrula
V Early somitogenesis
VI Mid somitogenesis
VII Late somitogenesis
VIII Early pharyngula
IX Mid pharyngula
X Late pharyngula
XI Hatchling
XII Larvae
Clusters
Astatotilapia calliptera (LM)
‡Curated annotation
E
Proportion of counts
Genome Feature
Exon
3' UTR
5' UTR
Intergenic
Intron (non-expressed gene)
Intron (expressed gene)

Fig. S2

Fig. S2. Additional data on TE expression dynamics during early development in African cichlids. (**A**) Number of TE families expressed in early development of Lake Malawi cichlids. The two panels above depict overall numbers or proportion of expressed families, while the panels below correspond to the number and proportion of expressed TE families by TE class. (**B**) Expression of TE families belonging to major TE classes throughout early development of *Tropheops* sp. 'mauve'. Expression in regularised log (rlog) normalised counts. Reads were mapped to the Lake Malawi reference genome (*A. calliptera*) and to non-curated (upper panel) and curated TE annotations (lower panel). (**C**) Expression of TE families across early cichlid development with different panels showing distinct TE superfamilies. Panels above depict DNA transposon superfamilies and panels below depict retrotransposon superfamilies. Expression shown as rlog normalised counts. P-values were calculated with Wilcoxon rank-sum tests (using Benjamini & Hochberg correction) comparing expression in *A. calliptera* versus *Tropheops sp. 'mauve'* in each developmental stage. Very few differences in expression are observed between *A. calliptera* and *Tropheops sp. 'mauve'*. Developmental stages: 2C, 2-cell; 1k, 1k cells; EG, early gastrulation; LG, late gastrulation; ES, early somitogenesis; MS, mid somitogenesis; LS, late somitogenesis; EP, early pharyngula; MP, mid pharyngula; LP, late pharyngula; HA, hatchling; LA, larval. Significance notation as follows: $*0.01 \le p$ value < 0.05; $**0.001 \le p$ value < 0.01; *** p-value < 0.001. (**D**) Heatmap showing differential expression and k-means clustering of individual TE loci in early stages of cichlid development. The four columns in each developmental stage represent different biological replicates. Analysis done as in Chang et al., 2022 [13], using SQuIRE counts mapped to the curated TE annotation of AC. Expression data represented as a z-score. (**E**) Stacked bar plot showing the proportion of counts in each library that correspond to TEs, which overlap with the indicated genome features. This analysis was conducted using the SQuIRE counts from AC samples, mapped to the AC genome and using the curated Lake Malawi TE annotation, and the publicly available AC gene annotation. Overlaps and categories were defined similarly to Chang et al., 2022 [13]. AC, *Astatotilapia calliptera*; LM, Lake Malawi; rlog, regularised log; TM, *Tropheops* sp. 'mauve'.

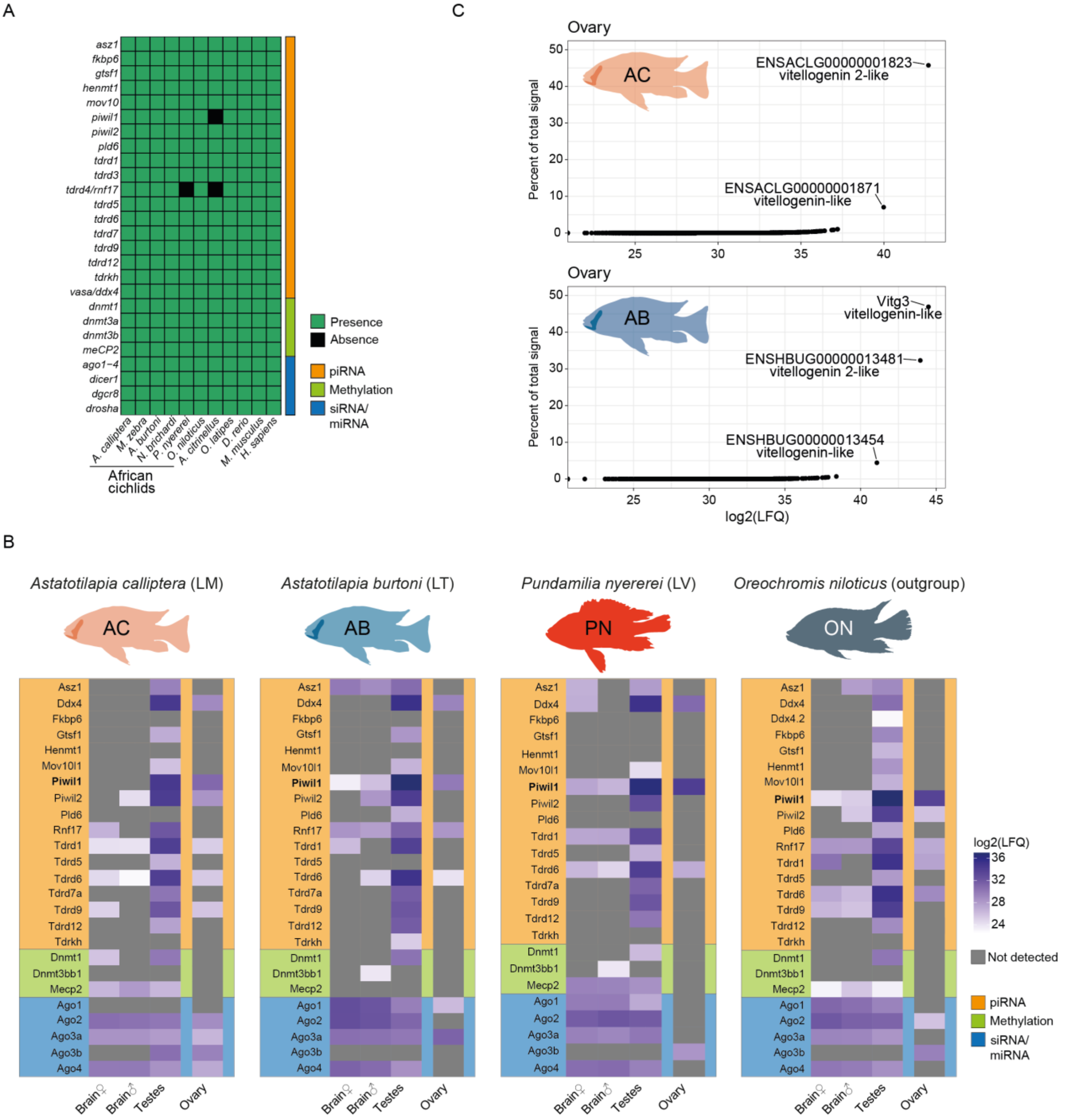

Fig. S3. Conservation and expression of epigenetic silencing factors in African cichlids. (**A**) Tileplot depicting conservation of epigenetic silencing factors in cichlids. All factors are conserved, besides three exceptions. (**B**) Label-free quantitative proteomics results showing expression of epigenetic silencing factors at the protein level. Only proteins detected at least in one organ in one species are shown. Proteins not detected in any of the species (TDRD3, TDRD7B, DNMT3AA, DNTM3AB, DNMT3BB2, DICER1, DGCR8, and DROSHA) are not shown. Ovary samples are shown in an isolated column to depict that direct comparisons with the other organs are to be avoided, as protein detection in ovaries was hampered by extremely abundant yolk proteins. (**C**) Extremely abundant yolk proteins are detected in label-free quantitative proteomics of ovaries (shown as percent of total LFQ signal), precluding robust detection of other proteins. AB, *Astatotilapia burtoni*; AC, *Astatotilapia calliptera*; LM, Lake Malawi; LT, Lake Tanganyika; LV, Lake Victoria; ON, *Oreochromis niloticus*; PN, *Pundamilia nyererei*.

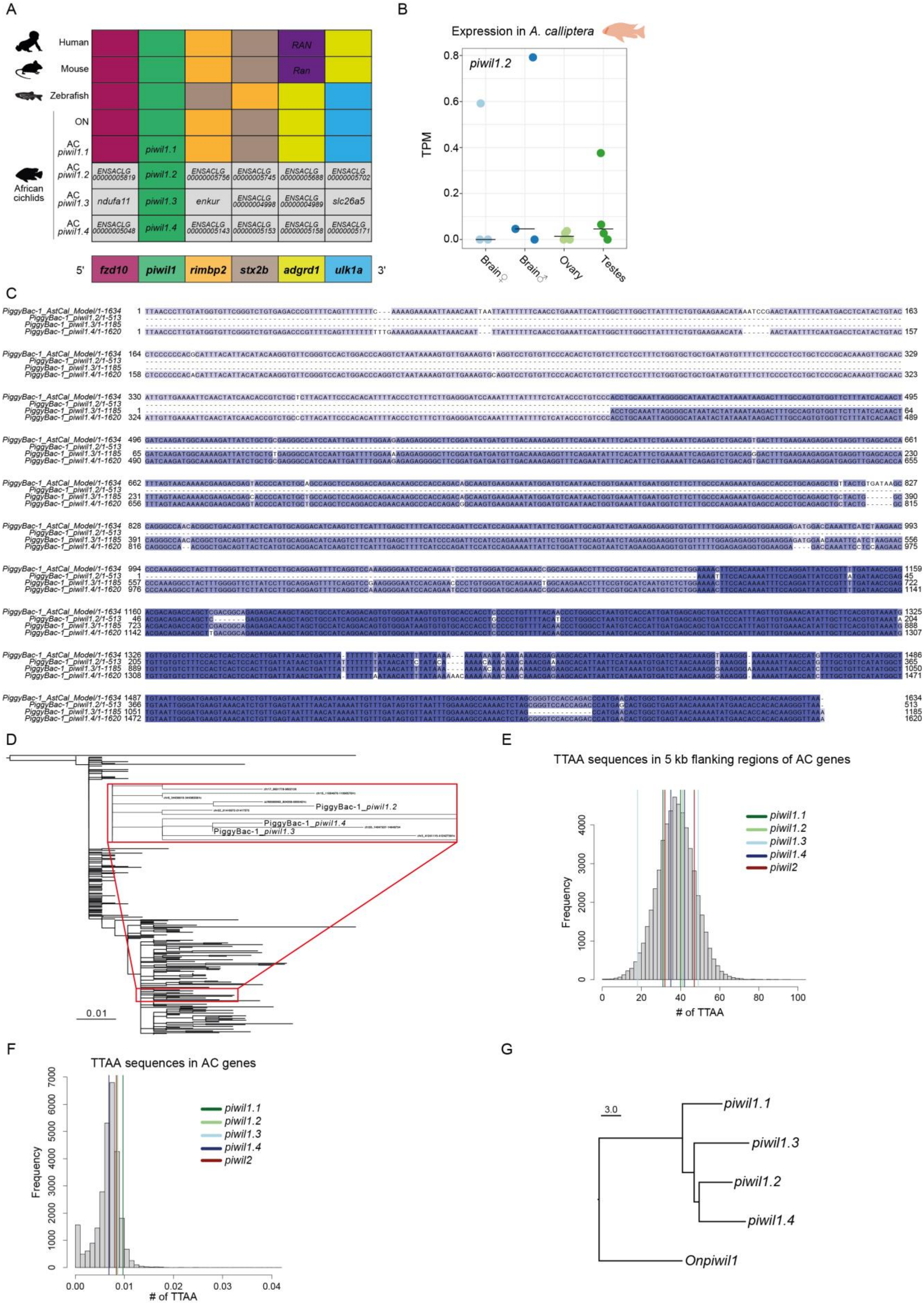
A
Human
Mouse
Zebrafish
ON
AC piwil1.1
AC piwil1.2
AC piwil1.3
AC piwil1.4
African cichlids
RAN
Ran
piwil1.1
ENSACLG 00000005819
piwil1.2
ENSACLG 00000005756
ENSACLG 00000005745
ENSACLG 00000005688
ENSACLG 00000005702
ndufa11
piwil1.3
enkur
ENSACLG 00000004998
ENSACLG 00000004989
slc26a5
ENSACLG 00000005048
piwil1.4
ENSACLG 00000005143
ENSACLG 00000005153
ENSACLG 00000005158
ENSACLG 00000005171
5'
fzd10
piwil1
rimbp2
stx2b
adgrd1
ulk1a
3'
B
Expression in A. calliptera
piwil1.2
TPM
0.8
0.6
0.4
0.2
0.0
Brain♀
Brain♂
Ovary
Testes
C
PiggyBac-1_AstCal_Model/1-1634
PiggyBac-1_piwil1.2/1-513
PiggyBac-1_piwil1.3/1-1185
PiggyBac-1_piwil1.4/1-1620
D
PiggyBac-1_piwil1.2
PiggyBac-1_piwil1.4
PiggyBac-1_piwil1.3
0.01
E
TTAA sequences in 5 kb flanking regions of AC genes
piwil1.1
piwil1.2
piwil1.3
piwil1.4
piwil2
Frequency
# of TTAA
F
TTAA sequences in AC genes
piwil1.1
piwil1.2
piwil1.3
piwil1.4
piwil2
Frequency
# of TTAA
G
3.0
piwil1.1
piwil1.3
piwil1.2
piwil1.4
Onpiwil1

Fig. S4

Fig. S4. Additional data on the expansion of *piwil1* genes in Lake Malawi cichlids. (**A**) Synteny analysis of *piwil1* genes in vertebrates. Each row of adjacent squares represents the order, oriented from 5' to 3', of genes adjacent to *piwil1* (in green). Squares of the same colour represent the same gene. The lower row of squares shows the consensus synteny and the correspondence between the colour-code and each gene. Grey squares indicate genes in linkage with only one *piwil1* gene. Of all *A. calliptera piwil1* genes, *piwil1.1* is the only gene syntenic with the *piwil1* gene of other vertebrates. AC, *Astatotilapia calliptera*; ON, *Oreochromis niloticus*. (**B**) Expression of *piwil1.2* in gonads and brain of *A. calliptera*, in Transcripts per Million (TPM). Data points represent distinct biological replicates. (**C**) Multiple sequence alignment of the PiggyBac-1 family consensus model of the curated *A. calliptera* TE library (sequence at the top) with the PiggyBac-1 sequences directly 3' of *piwil1.2*, *piwil1.3*, and *piwil1.4*. Colouring is according to sequence identity. According to open reading frame predictions, the PiggyBac-1 family is not expected to encode a full-length transposase. Additional mutations have accumulated in *piwil1*-associated PiggyBac-1 TEs. (**D**) Phylogenetic tree constructed from all PiggyBac-1 TEs in the *A. calliptera* genome that align with *piwil1*-associated PiggyBac-1 TEs. Tree was rooted at the midpoint. The branches with the *piwil1*-associated PiggyBac-1 TEs are shown in detail in the red box inset, illustrating their relatedness. (**E**) Distribution of the number of TTAA sequences in the 5 kilobase flanking regions of all annotated genes in the *A. calliptera* (AC) genome. The number of TTAA in the flanking regions of *piwi* genes is indicated in the figure with the coloured lines. The flanking regions of *piwi* genes do not have significantly different numbers of TTAA sequences versus the flanking regions of all other genes (Welch's t-test, p-value = 0.86). (**F**) Distribution of the number of TTAA sequences within all annotated genes (including introns) in the *A. calliptera* (AC) genome. The number of TTAA sequences within *piwi* genes is highlighted in the distribution with the coloured lines. *piwi* genes do have slightly higher TTAA numbers than all other genes (Welch's t-test, p-value = 0.018). (**G**) Neighbour-joining tree representing the Hamming distance between the coding sequences within the region shared by all *A. calliptera piwil1* genomic sequences (Region S in **Figure 2A**). The equivalent region in *piwil1* of *O. niloticus* (*Onpiwil1*) is included as an outgroup.

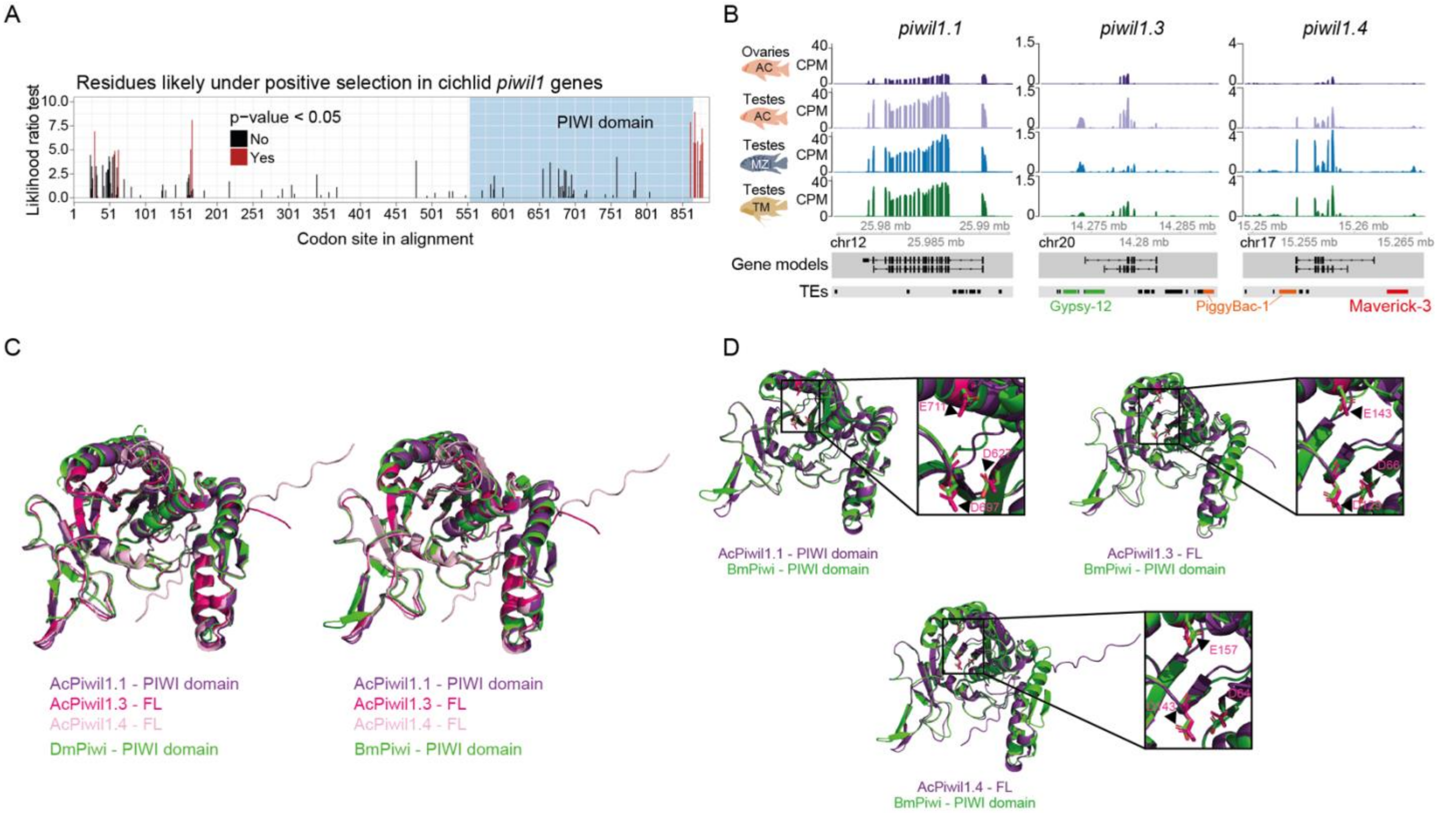

Fig. S5. Expression, function, and evolution of cichlid *piwil1* genes. (**A**) Residues very likely to be under positive selection according to Mixed Effects Model of Evolution (MEME) [98] across the entire alignment of African cichlid Piwil1 coding sequences. A region in the C-terminal portion of the proteins has 8 amino acid residues predicted to be under positive selection. See additional analysis in Additional File 2: Table S1. (**B**) Genome tracks showing the mRNA expression of *piwil1.1* (left panel), *piwil1.3* (central panel), and *piwil1.4* (right panel). Large TE fragments flanking *piwil1.3* and *piwil1.4* are annotated and coloured in the TE track. mRNA expression shown in Counts per Million (CPM). (**C**) Structural alignments of the AlphaFold-predicted PIWI domain of *Astatotilapia calliptera* (Ac) Piwil1.1, or full-length Piwil1.3 and Piwil1.4, with the experimentally determined structures of the PIWI domains of *Drosophila melanogaster* (Dm) Piwi (left) and *Bombyx mori* (Bm) Siwi (right). (**D**) Structural alignments of the PIWI domain of Bombyx mori (Bm) Siwi protein with the AlphaFold predictions of the Piwil1.1 (using only PIWI domain), Piwil1.3 (full-length), and Piwil1.4 (full-length) of *A. calliptera*. Insets focus on the regions with the integral residues of the catalytic triad, which are indicated with black or white arrowheads. AC, *Astatotilapia calliptera*; MZ, *Maylandia zebra*; TM, *Tropheops* sp. ‘mauve’.

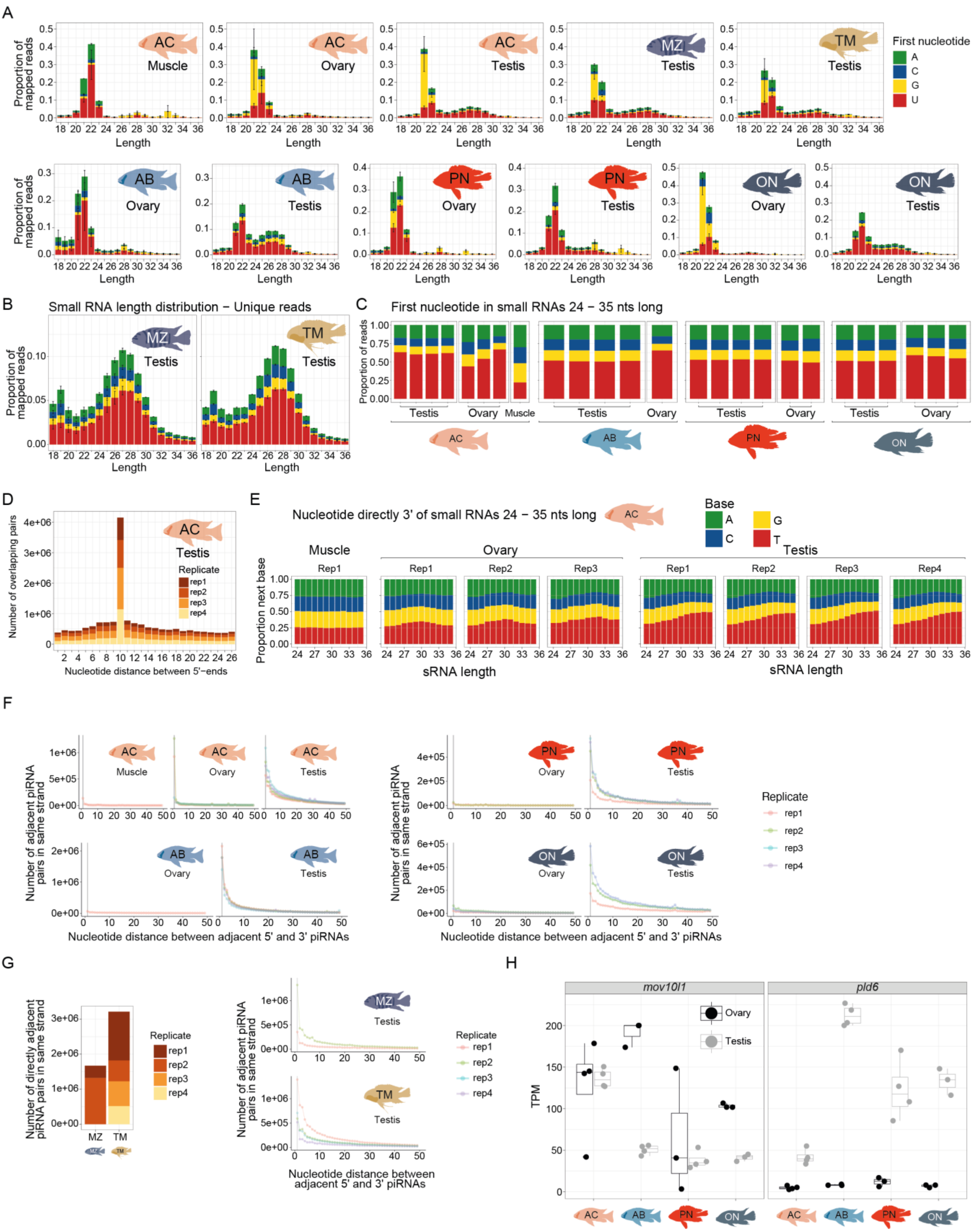

A
Proportion of mapped reads
Length
First nucleotide
A
C
G
U
AC Muscle
AC Ovary
AC Testis
MZ Testis
TM Testis
AB Ovary
AB Testis
PN Ovary
PN Testis
ON Ovary
ON Testis
B
Small RNA length distribution − Unique reads
C
First nucleotide in small RNAs 24 − 35 nts long
Proportion of reads
D
Number of overlapping pairs
Replicate
rep1
rep2
rep3
rep4
Nucleotide distance between 5'−ends
E
Nucleotide directly 3' of small RNAs 24 − 35 nts long
Base
T
Muscle
Ovary
Testis
Rep1
Rep2
Rep3
Rep4
Proportion next base
sRNA length
F
Number of adjacent piRNA pairs in same strand
Nucleotide distance between adjacent 5' and 3' piRNAs
G
Number of directly adjacent piRNA pairs in same strand
Nucleotide distance between adjacent 5' and 3' piRNAs
H
mov10l1
pld6
TPM

Fig. S6

Fig. S6. Profile and sequence signatures of sRNAs in African cichlids. (**A**) sRNA length distribution profiles of all reads (without collapsing into unique reads) display prominent peaks at 21-22 nucleotides, likely attributable to abundant microRNAs. Colours display the identity of the first nucleotide in the sRNA. (**B**) sRNA length distribution profiles of unique reads in *M. zebra* and *T.* sp. 'mauve' testes. Identity of first nucleotide is indicated by the colouring, which is colour coded as in (A). (**C**) First nucleotide identity in sRNAs 24-35 nucleotides long. Same colouring by first nucleotide as in (A). (**D**) Number of overlapping sRNA read pairs according to the length of the overlap in *A. calliptera* testis replicates. The peak at 10 nucleotides supports a ping-pong signature. (**E**) Identity of nucleotide directly 3' of the last nucleotide of sRNAs 24-35 nucleotides long. A bias for a T is consistent with a signature of phased piRNA biogenesis. (**F**) Number of directly adjacent piRNA pairs and the distance separating them. Grey line indicates distance of 1 nucleotide, indicative of phased piRNA biogenesis, where two piRNAs are produced consecutively. Testes of all species have high numbers of piRNA pairs at 1 nucleotide distance, but this signature is not clear in ovaries, except the ovaries of AC. (**G**) On the right, plots as in (F) showing the number of piRNA pairs and the distance between each member of the pair. Grey line marks distance of 1 nucleotide. On the left, histogram with the number of piRNA pairs distant 1 nucleotide from each other (equivalent to the grey line in the plots on the right). (**H**) Expression of *mov10l1* and *pld6* in gonads of representative East African cichlids, in Transcripts per Million (TPM). Data points represent distinct biological replicates. AB, *Astatotilapia burtoni*; AC, *Astatotilapia calliptera*; MZ, *Maylandia zebra*; ON, *Oreochromis niloticus*; PN, *Pundamilia nyererei*; TM, *Tropheops* sp. 'mauve'; sRNA, small RNA.

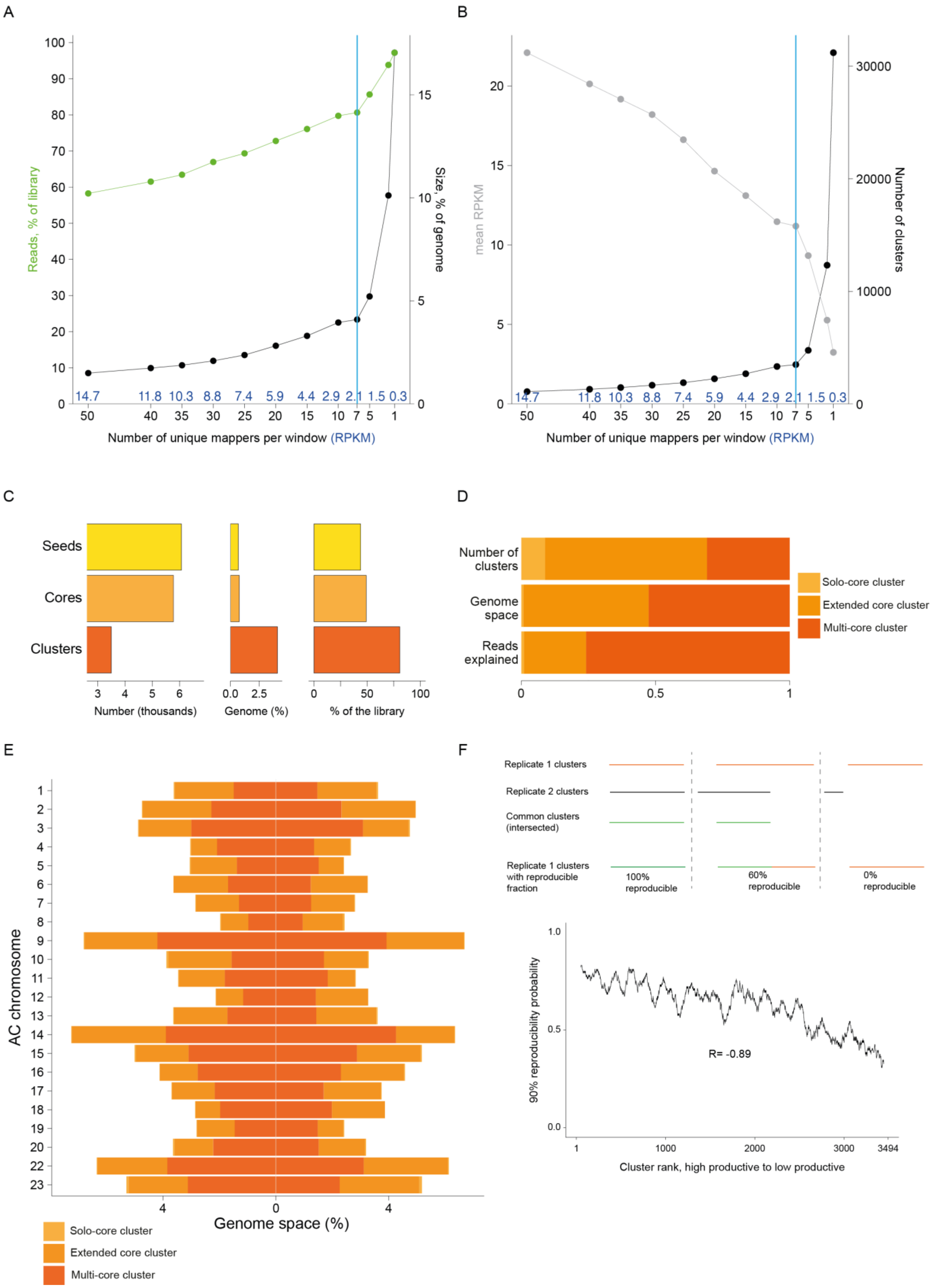
A
Reads, % of library
Size, % of genome
Number of unique mappers per window (RPKM)
B
mean RPKM
Number of clusters
Number of unique mappers per window (RPKM)
C
Seeds
Cores
Clusters
Number (thousands)
Genome (%)
% of the library
D
Number of clusters
Genome space
Reads explained
Solo-core cluster
Extended core cluster
Multi-core cluster
E
AC chromosome
Genome space (%)
Solo-core cluster
Extended core cluster
Multi-core cluster
F
Replicate 1 clusters
Replicate 2 clusters
Common clusters (intersected)
Replicate 1 clusters with reproducible fraction
100% reproducible
60% reproducible
0% reproducible
90% reproducibility probability
R= -0.89
Cluster rank, high productive to low productive

Fig. S7

Fig. S7. Benchmarking piRNA Cluster Builder (piCB) parameters. (**A-B**) Optimisation curves for piCB clustering parameters in one library from *A. calliptera* testes. The x axis shows the threshold number of uniquely mapping reads required per default sliding window of 350 nt (in black) and corresponding Reads per Kilobase Million (RPKM, in blue). The default parameters of piCB are set at 7 unique mappers per windows (equivalent to 2 RPKM) and is indicated in (A-B) as blue vertical lines. In (A), the left Y axis indicates the fraction of the reads in the library explained by the identified clusters (green line), while the right Y axis indicates the percentage of the reference genome occupied by the identified clusters (black line). In (B), the left Y axis indicates the mean RPKM (grey line), and the right Y axis indicates the number of clusters identified (black line). The default parameters were reasoned to perform adequately and were used in subsequent analysis. (**C**) Characterization of seeds, cores, and clusters assembled from piRNAs from one small RNA library from *A. calliptera* testes. The three panels show, from left to right: the number in thousands of each category, the percentage of genome occupied by each category, and the percentage of library attributable to each category. (**D**) Proportion of clusters of each type (solo-core, extended core, and multi-core as defined in ref. [61]) according to their number, genome space occupied, and fraction of library reads explained. Multi-core clusters are a minority, but correspond to approximately half of the genomic space of all clusters, explaining the majority of the reads. (**E**) Chromosomal distribution of piRNA clusters in *A. calliptera* testes according to genome space occupied and the type of cluster identified by piCB. (**F**) The upper panel is a scheme depicting the strategy used to address reproducibility of the clusters. Panel below shows the proportion of clusters that are at least 90% reproducible across their whole length, according to the cluster rank in terms of productivity. This figure shows that most of the clusters are highly reproducible, and cluster productivity affects this. We performed an additional analysis addressing reproducibility of clusters by comparing the clusters in two *A. calliptera* testis small RNA libraries to a random process. We did this by computing the ratio of common (intersection of the two replicates) genomic space length to reduced (cluster in either of the replicates) genomic space length, using a bootstrapping approach. Bootstrapping was conducted by relocating the clusters to random coordinates and strand, while keeping the number and length of the clusters. This analysis supported a non-random intersection between the replicates (p-value $<10^{-4}$).

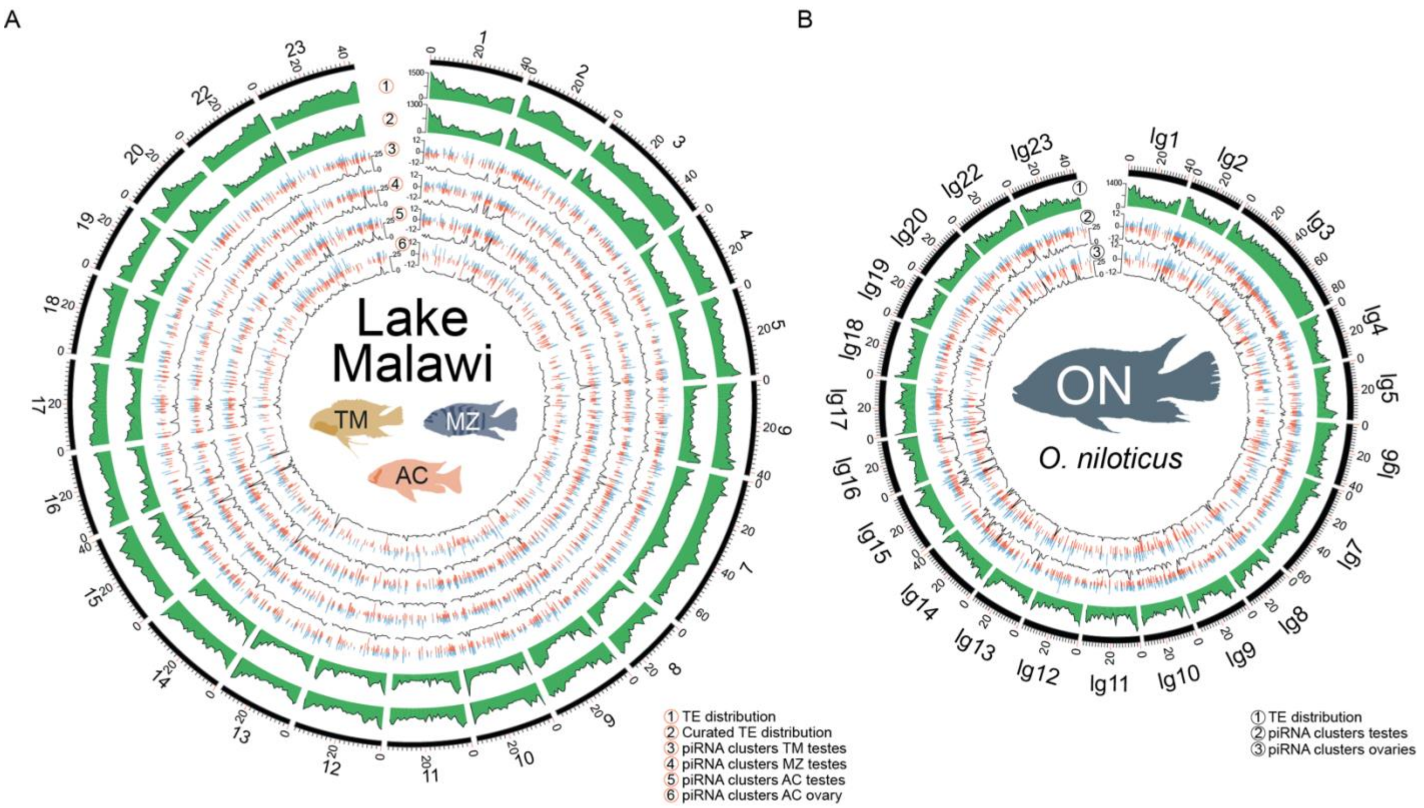

Fig. S8. Genomic location of piRNA clusters in African cichlids. (**A**) Circos plot showing TE distributions (tracks 1-2) from non-curated (track 1) and curated (track 2) annotations and chromosomal locations of piRNA clusters in Lake Malawi cichlid gonads (tracks 3-6). In tracks 3-6, blue and red represent the log2 mean Reads Per Kilobase Million (RPKM) of piRNA clusters in the plus and minus strands, respectively. In the bottom of tracks 3-6 is a line plot with the density of clusters. (**B**) Circos plot showing TE distribution (track 1), and the chromosomal locations of piRNA clusters in testes (track 2), and ovaries (track 3). In tracks 1-2, blue and red represent the log2 mean RPKM of piRNA clusters in the plus and minus strands, respectively. In the bottom of tracks 2-3 is a line plot with the density of clusters.

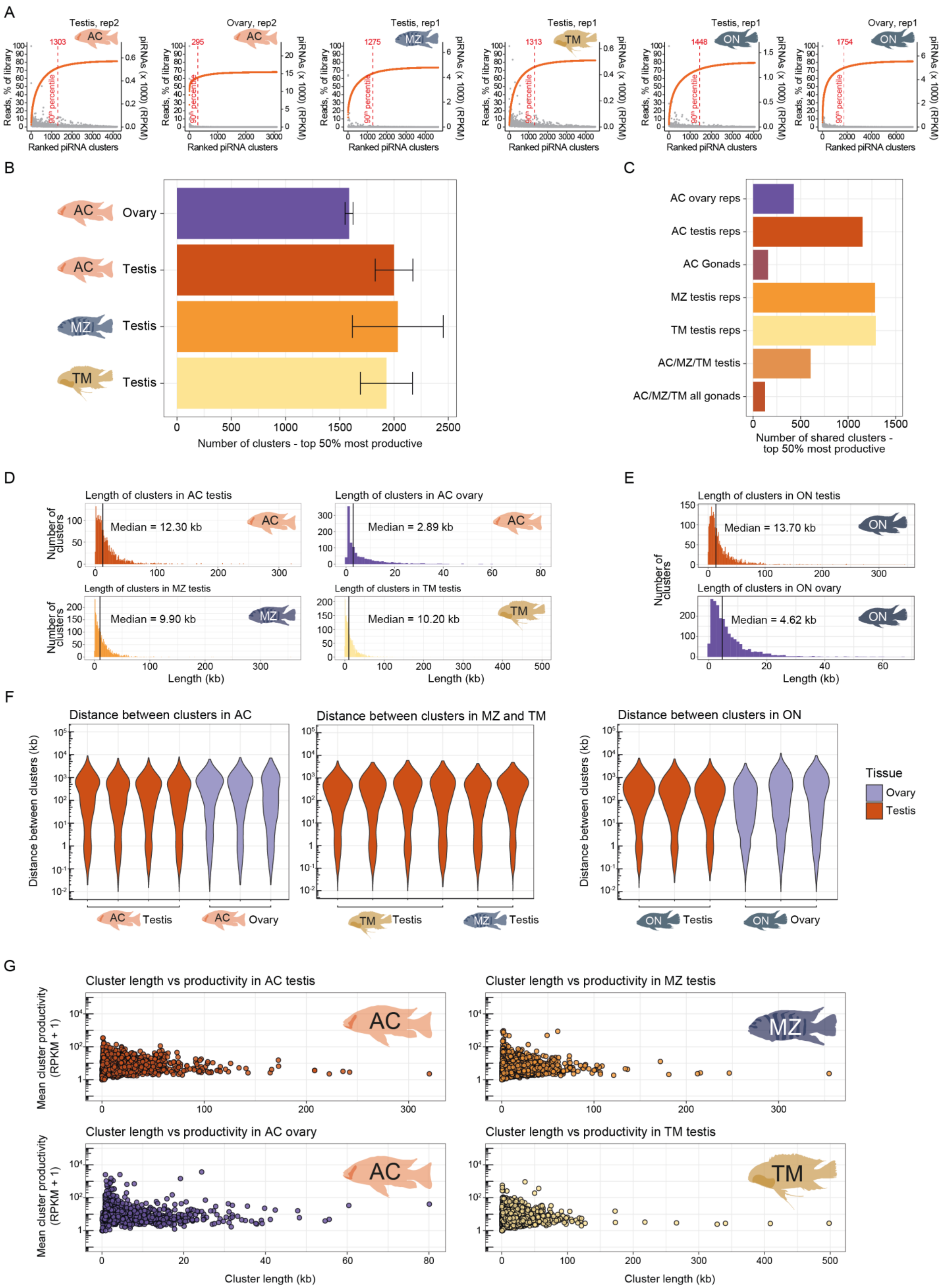
A
Testis, rep2
AC
1303
90th percentile
Reads, % of library
piRNAs (x 1000) (RPKM)
Ranked piRNA clusters
Ovary, rep2
295
Testis, rep1
MZ
1275
TM
1313
ON
1448
Ovary, rep1
1754
B
AC Ovary
AC Testis
MZ Testis
TM Testis
Number of clusters - top 50% most productive
C
AC ovary reps
AC testis reps
AC Gonads
MZ testis reps
TM testis reps
AC/MZ/TM testis
AC/MZ/TM all gonads
Number of shared clusters - top 50% most productive
D
Length of clusters in AC testis
Median = 12.30 kb
Length of clusters in AC ovary
Median = 2.89 kb
Length of clusters in MZ testis
Median = 9.90 kb
Length of clusters in TM testis
Median = 10.20 kb
Number of clusters
Length (kb)
E
Length of clusters in ON testis
Median = 13.70 kb
Length of clusters in ON ovary
Median = 4.62 kb
Number of clusters
Length (kb)
F
Distance between clusters in AC
Distance between clusters in MZ and TM
Distance between clusters in ON
Distance between clusters (kb)
AC Testis
AC Ovary
TM Testis
MZ Testis
ON Testis
ON Ovary
Tissue
Ovary
Testis
G
Cluster length vs productivity in AC testis
Cluster length vs productivity in MZ testis
Cluster length vs productivity in AC ovary
Cluster length vs productivity in TM testis
Mean cluster productivity (RPKM + 1)
Cluster length (kb)

Fig. S9

Fig. S9. Additional features of cichlid piRNA clusters. (**A**) Ranking of piRNA clusters according to productivity across Lake Malawi cichlids and *O. niloticus*. Cumulative fraction shown as an orange line. Dashed red line indicates the 90th percentile of piRNA production and the number of clusters producing 90% of piRNA reads in clusters. (**B**) The mean number of clusters identified in Lake Malawi cichlid gonads, including only the top 50% most productive clusters. Error bars represent standard deviation. (**C**) The number of clusters shared between the replicates of the organs indicated. These intersections were calculated using the top 50% most productive clusters. (**D**) Histograms showing the length distribution of piRNA clusters in *A. calliptera* (two upper panels) *M. zebra* (lower left panel) and *T.* sp. 'mauve' (lower right panel). Vertical black lines denote the median length. (**E**) Histogram showing the distribution of the lengths of piRNA clusters in *O. niloticus*. Vertical black lines denote the median length. (**F**) Distance between clusters, in kilobase (kb), in each replicate of the indicated organs and species. Left panel for *A. calliptera* gonads, central panel for *T.* sp. 'mauve' and *M. zebra* testes, and right panel for *O. niloticus* gonads. (**G**) Relationship between piRNA cluster length and cluster productivity. Productivity is plotted as mean Reads Per Kilobase Million (RPKM) of a cluster across all replicates. AC, *Astatotilapia calliptera*; MZ, *Maylandia zebra*; ON, *Oreochromis niloticus*; TM, *Tropheops* sp. 'mauve'.

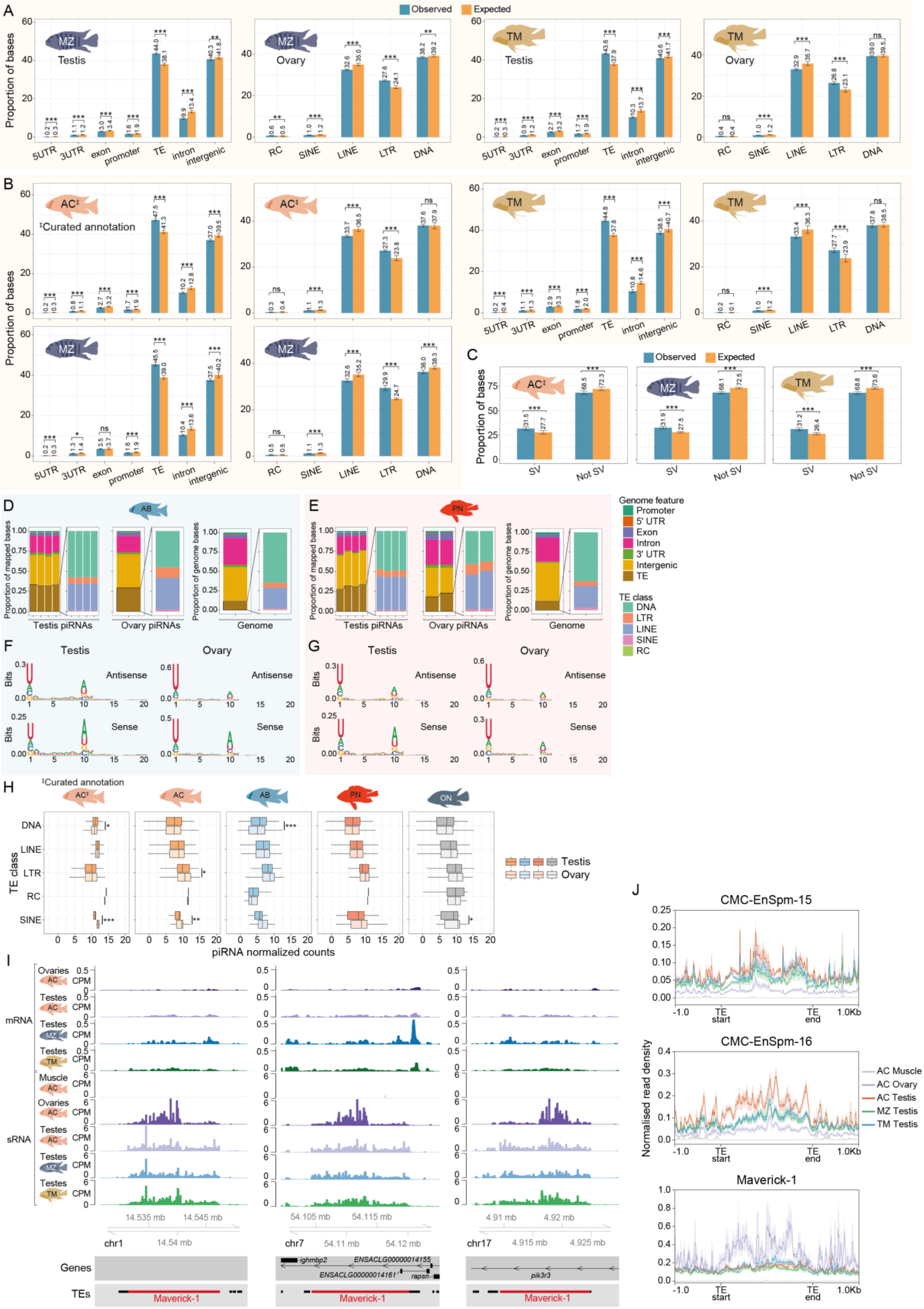
A
Observed
Expected
Proportion of bases
MZ
Testis
Ovary
TM
5UTR
3UTR
exon
promoter
TE
intron
intergenic
RC
SINE
LINE
LTR
DNA
B
AC‡
‡Curated annotation
C
SV
Not SV
D
AB
E
PN
Proportion of mapped bases
Proportion of genome bases
Testis piRNAs
Ovary piRNAs
Genome
Genome feature
Promoter
5' UTR
Exon
Intron
3' UTR
Intergenic
TE
TE class
DNA
LTR
LINE
SINE
RC
F
G
Bits
Antisense
Sense
H
‡Curated annotation
AC
ON
TE class
piRNA normalized counts
Testis
Ovary
I
mRNA
sRNA
Ovaries
Testes
Muscle
CPM
14.535 mb
14.545 mb
chr1
14.54 mb
54.105 mb
54.115 mb
chr7
54.11 mb
54.12 mb
4.91 mb
4.92 mb
chr17
4.915 mb
4.925 mb
Genes
TEs
ighmbp2
ENSACLG00000014155
ENSACLG00000014161
rapsn
pik3r3
Maverick-1
J
CMC-EnSpm-15
CMC-EnSpm-16
Maverick-1
Normalised read density
TE start
TE end
-1.0
1.0Kb
AC Muscle
AC Ovary
AC Testis
MZ Testis
TM Testis

Fig. S10

Fig. S10. The genomic origin and sequence signatures of piRNAs targeting TEs in cichlids. (**A**) Observed and expected values at genomic features and TE classes that piRNA clusters overlap with in Lake Malawi cichlids MZ and TM. (**B**) The genomic composition of the diverging piRNA clusters in Lake Malawi cichlids, present in one species (AC, MZ, or TM), but not in at least one of the other species. The species where the diverging clusters is present is indicated in the panel. In (B), TE features used to calculate overlaps with piRNA clusters correspond to the curated TE annotation. (**C**) Proportion of mapped bases of species-variable piRNA clusters that overlap with structural variants (SVs) in the Lake Malawi pangenome, defined in Quah et al., 2024 [5]. The species where the variable clusters are present is indicated in the panel. (**D-E**) Genomic features that 24-35 nucleotide long piRNAs map to in *A. burtoni* (D) and *P. nyererei* (E). Each bar represents a separate replicate. Inset barplots specify the proportions of major TE classes that piRNAs map to. Genome bars represent the proportion of bases corresponding to genome-wide specific features and TE classes. (**F-G**) Sequence logos of 24-35 nucleotide long piRNAs mapping sense or antisense in regard to TE orientation in *A. burtoni* (F) and *P. nyererei* (G). (**H**) Expression of piRNAs mapping to the major TE classes. P-values were calculated with Wilcoxon rank-sum tests (using Benjamini & Hochberg correction) comparing expression in ovaries and testes for each TE class. We note that incomplete LTR annotation may overestimate the expression of LTR-mapping piRNAs (compare AC curated versus non-curated annotation). (A-C, H) Significance notation as follows: *0.01 ≤ p value < 0.05; **0.001 ≤ p value < 0.01; *** p-value < 0.001. (**I**) Genome tracks with the mRNA expression and 24-35 nucleotide sRNAs mapping to large Maverick TEs in Lake Malawi cichlids. mRNA and sRNA expression shown in Counts per Million (CPM). In the TE track, a curated TE annotation is shown, with the large Maverick element coloured in red. **(J)** Metagene plots depicting mean piRNA levels mapping to TEs of likely transpositionally active families (CMC-EnSpm-15, CMC-EnSpm-16, and Maverick-1). The shading represents standard error of replicates. TE start and TE end indicate start and end coordinates, respectively, of TE in the annotation. AB, *Astatotilapia burtoni*; AC, *Astatotilapia calliptera*; CPM, Counts per Million; MZ, *Maylandia zebra*; ns, not statistically significant; PN, *Pundamilia nyererei*; SV, structural variant; TM, *Tropheops* sp. ‘mauve’; sRNA, small RNA.

A

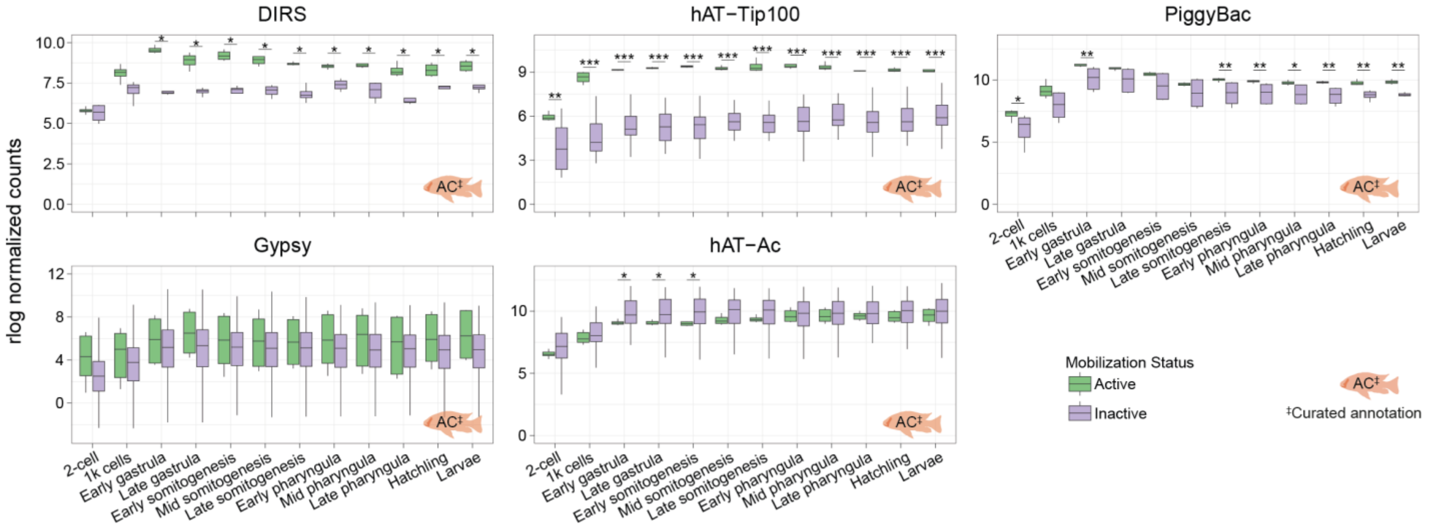

Fig. S11. Additional data on the expression of TE families in early development according to likely transpositional status. (**A**) Regularised log (rlog) expression of TE families likely to be transpositionally active versus inactive, of the indicated superfamilies (DIRS, hAT-Tip100, PiggyBac, Gypsy, and hAT-Ac), throughout early development of *A. calliptera*. P-values were calculated with Wilcoxon rank-sum tests (using Benjamini & Hochberg correction) comparing expression of likely active versus inactive TE families in each developmental stage. A curated annotation of Lake Malawi TEs was used to calculate expression data. AC, *Astatotilapia calliptera*; rlog, regularised log. Significance notation as follows: *0.01 ≤ p value < 0.05; **0.001 ≤ p value < 0.01; *** p-value < 0.001.
